# Comparative analysis of feature-based ML and CNN for binucleated erythroblast quantification in myelodysplastic syndrome patients using imaging flow cytometry data

**DOI:** 10.1038/s41598-024-59875-x

**Published:** 2024-04-23

**Authors:** Carina A. Rosenberg, Matthew A. Rodrigues, Marie Bill, Maja Ludvigsen

**Affiliations:** 1 Department of Hematology, Aarhus University Hospital, Palle Juul-Jensens Boulevard 35, C115, 8200 Aarhus C, Denmark; 2 Amnis Flow Cytometry, Cytek Biosciences, Seattle, WA USA; 3 Department of Clinical Medicine, Aarhus University, Aarhus, Denmark

**Keywords:** Imaging flow cytometry, Artificial intelligence, Feature-based machine learning, Convolutional neural network, Dyserythropoiesis, Myelodysplastic syndrome, Myelodysplastic syndrome, Preclinical research

## Abstract

Myelodysplastic syndrome is primarily characterized by dysplasia in the bone marrow (BM), presenting a challenge in consistent morphology interpretation. Accurate diagnosis through traditional slide-based analysis is difficult, necessitating a standardized objective technique. Over the past two decades, imaging flow cytometry (IFC) has proven effective in combining image-based morphometric analyses with high-parameter phenotyping. We have previously demonstrated the effectiveness of combining IFC with a feature-based machine learning algorithm to accurately identify and quantify rare binucleated erythroblasts (BNEs) in dyserythropoietic BM cells. However, a feature-based workflow poses challenges requiring software-specific expertise. Here we employ a Convolutional Neural Network (CNN) algorithm for BNE identification and differentiation from doublets and cells with irregular nuclear morphology in IFC data. We demonstrate that this simplified AI workflow, coupled with a powerful CNN algorithm, achieves comparable BNE quantification accuracy to manual and feature-based analysis with substantial time savings, eliminating workflow complexity. This streamlined approach holds significant clinical value, enhancing IFC accessibility for routine diagnostic purposes.

## Introduction

Myelodysplastic syndrome (MDS) is characterized as a highly heterogeneous group of clonal stem cell disorders marked by a range of different dysplastic changes in the bone marrow (BM) together with disrupted hematopoiesis, varying degrees of cytopenias, and an increased risk of progression to acute myeloid leukemia (AML)^1–5^. In the diagnostic setting, morphologic BM dysplasia together with persistent cytopenia remain the hallmarks of MDS^5,6^. However, accurate diagnosis of cases in which mild cytopenias and subtle dysplastic changes are present can be difficult, and inter-scorer variability and subjectivity may be present, even among experienced hematopathologists^1,3,7,8^. Furthermore, in many cases qualifying dysplasia is not identified, and indefinite conclusions may be arrived at despite the presence of persistent cytopenias, categorizing patients into two streams: idiopathic cytopenia of undetermined significance (ICUS)^9^ or, when evidence of clonality is present, clonal cytopenia of undetermined significance (CCUS)^10^. Interestingly, carrying certain mutational patterns leads to similar overall survival and risk of disease progression for high-risk CCUS and low-risk MDS patients, thus suggesting that such high-risk CCUS cases should be classified as low-risk MDS^11^. As a result of this diagnostic complexity, high-throughput, objective, standardizable and reproducible methods that permit distinction of MDS from non-clonal reactive causes of cytopenia and dysplasia are desirable. Moreover, rare events that are indicative of MDS such as binucleated erythroblasts (BNEs), while easy to identify using visual microscopy can be challenging to quantify in large numbers, thus limiting statistical robustness.

Imaging flow cytometry (IFC) using the ImageStream^®^X MKII (ISX) may be able to address this, as it combines high-throughput data acquisition capacity and statistical robustness of conventional multicolor flow cytometry (MFC) together with high-resolution imaging capabilities of microscopy in a single system. The ISX permits simultaneous capture of 12 images (2 brightfield (BF) and 10 fluorescent) from every individual cell that passes through the system^12^. The acquired image data from thousands of cells may be analyzed using traditional MFC gating strategies to identify phenotypical markers, as well as applying feature-based algorithms and artificial intelligence to quantify morphometric changes that can be standardized and automated, reducing subjectivity and scorer variability^13,14^.

In recent years, several publications have illustrated the relevance and potential of IFC to supplement and perhaps enhance traditional visual microscopic techniques^15,16^. In the clinical space, applications such as phenotypical and morphological changes in red blood cell structure in sickle cell disease^17–19^, hereditary spherocytosis^20^, red blood cell storage lesions^21,22^, phenotypic blast heterogeneity^23^, assessment of leukocyte-platelet aggregates^24^, detection of numerical and structural chromosomal abnormalities^25–27^, and detection of cytoplasmic nucleophosmin in NPM1 mutated AML patients^28,29^ show the potential strength of IFC. Recently, we applied the IFC technology to show that dysmorphometric changes in the erythroid cell lineage in MDS BM samples with known dyserythropoiesis could be identified and quantified both phenotypically and morphometrically. We also applied a newly available feature-based machine learning (FBML) algorithm to identify distinct image morphologies present in rare BNEs to maximize accuracy in their identification and quantification^30^.

While feature-based analysis is the gold standard method to examine IFC data, generating effective and appropriate analysis strategies can be extremely challenging, time-consuming and ineffective given the rigidity of image segmentation^22,31^. Therefore, AI-based approaches are rapidly gaining ground^32–35^ based on their ability to examine information from multiple levels in image data. As such, convolutional neural networks (CNNs) can identify subtleties in complex image morphologies and are able to discriminate populations with more flexibility and accuracy than feature-based analysis^36,37^. However, one significant disadvantage regarding the use of AI algorithms has been the reliance on computer scientists for development, optimization and validation due to the advanced coding knowledge required for implementation. To address this challenge, the Amnis^®^ AI (AAI) software package was developed with a convenient graphical user interface to allow researchers without advanced coding skills to directly develop, train, and validate CNN models to analyze IFC image data. This new software package has been used recently to differentiate silicone oil droplets from protein aggregates^38^, to quantify micronuclei in genetic toxicology^14^, and to assess boar sperm acrosome health^39^.

In this paper, using the raw data from our previous work^30^, we examine the use of the AAI software to identify BNEs and differentiate them from doublet events and other non-BNE images. We compare the AAI-derived BNE frequencies to results previously obtained using FBML and illustrate their similarities. Importantly, the imagery of every individual cell, from both patients and controls, was manually examined to visually detect and validate the genuineness of the BNEs. Ultimately, we show that an AI-based analysis is more straightforward to construct and implement in comparison to traditional feature-based analysis. We believe that our AI-driven approach has the potential to both improve and streamline work procedures in clinical laboratories which is of major importance as limited resources in our health care system calls for implementing less labor-intensive methods.

## Methods

### Image data

For this study, we leverage the IFC imagery and raw data acquired in our prior study^30^. The dataset was comprised of BM samples from 14 MDS patients, six ICUS/CCUS patients, six non-MDS controls, and 11 healthy controls. The selection of MDS and control BM samples, along with sample preparation, data acquisition, gating procedure for erythroblast subpopulations, and FBML quantification of BNEs, were extensively described previously^30^. The Central Denmark Region Committee on Health Research Ethics (record no.: 1-10-72-125-17) and the Danish Data Protection Agency (record no.: 1-16-02-849-17) approved the study.

### FBML model development using IDEAS machine learning module

The current study extends our prior research on BNE identification and quantification, leveraging data and processing procedures from our previous investigations^30^. In summary, we utilized the IDEAS-based ML module (version 6.3.17) in our forgoing study to differentiate BNEs from doublets and erythroblasts exhibiting irregular nuclear shapes. The ML module integrated within IDEAS generates masks and extracts morphometric features on a per object basis. The features are combined through Linear Discriminant Analysis, with weighting determined by user-defined truth populations manually tagged. Subsequently, the module identifies images within the broader dataset resembling the designated truth populations and assigns a classifier-specific value to each image, which can be visualized trough histograms and bivariate plots, making these ML classifiers versatile across different datasets. To utilize the ML module, a single file containing a minimum of 25 hand-tagged images is required for operation. Given the low frequency of BNEs and doublets within individual MDS patients and controls, we combined multiple files to ensure an adequate number of truth events for generating a robust classifier. A total of seven data files from MDS patients were combined. A BNE mask in combination with a Spot Count feature was used to detect candidate erythroblasts with two nuclei (2N). Finally, FBML models were applied to the 2N population and used to differentiate BNEs from doublets and erythroblasts with irregular nuclear shape in all patients and healthy control^30^.

### CNN model development using Amnis^®^ AI (AAI)

**Figure 1:**
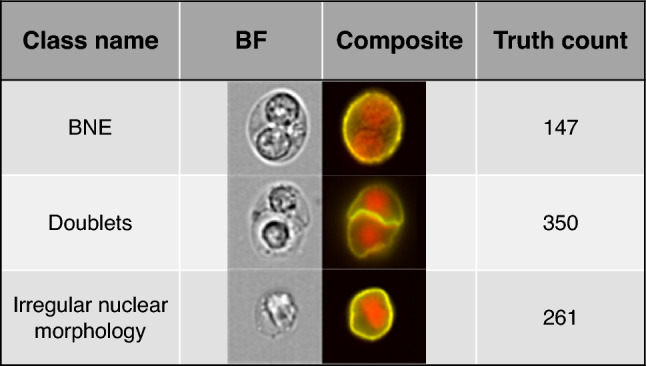
Data overview. Representative IFC imagery of the truth populations and key events that must be distinguished for correct BNE quantification. The key events include BNEs (n = 147), doublets (n = 350), and erythroblasts with irregular nuclear morphology (n = 261). Representative BF and nuclear imagery are shown, the latter as composite with CD235a PE staining overlaid onto the images.

**Figure 2:**
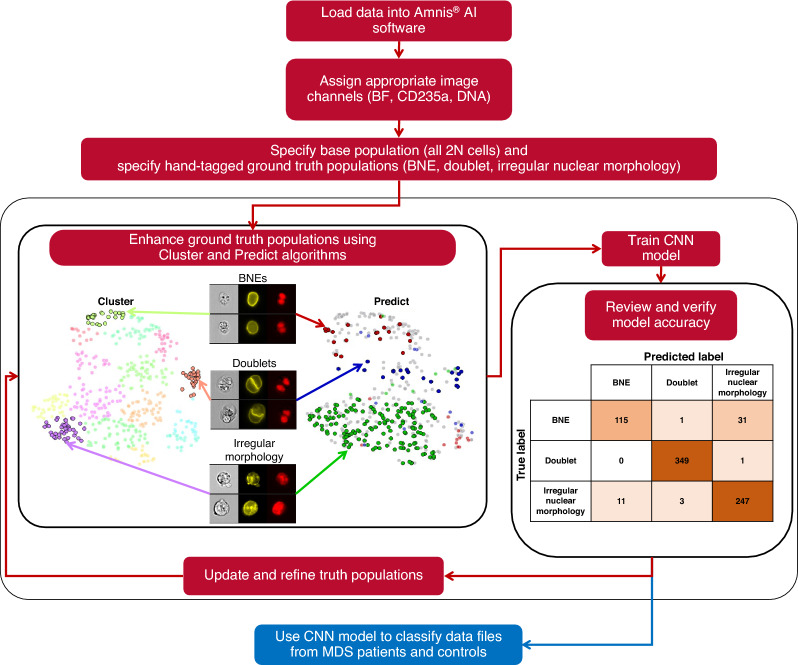
Diagrammatic view of the Amnis AI software workflow. Images from IFC data files were loaded into the AAI software using the available step-by-step wizard. Next, appropriate channels were assigned (i.e., BF, CD235a, and DNA), the base population (i.e., 2N cells, n = 758) was specified. Then ground truth populations were defined using the Cluster and Predict algorithms. The CNN model was trained, accuracy was reviewed, truth populations were updated and refined by assigning misclassified images to the appropriate model class, and the model was retrained. Finally, the CNN model was used to classify data files from MDS patients and controls.

To ensure a similar starting point in this work the 2N population from the combined data file was loaded into the AAI software (v2.0.7; Cytek Biosciences, Seattle, WA) that uses the Keras Application Programming interface v2.1.5 with Tensorflow v1.7.0 library and the VGG16 network to train a CNN and subsequently classify objects from relevant data files. Users interact with the AAI software through a straightforward graphical user interface (GUI), negating advanced coding requirements. A CNN model was trained to classify erythroblast nuclei into one of three model classes—BNE, doublet, or irregular nuclear morphology—using only the BF, CD235, and DRAQ5 images (Fig. 1). A total of 758 candidate BNE objects, identified by the “BNE spot count” feature in the IDEAS^®^ gating strategy^30^, were imported as the base population. To assign each object to the most appropriate model class prior to training, we used the Cluster and Predict algorithms (Fig. 2). The Cluster algorithm examines an automatically created segment of 1500 random objects from the base population, and groups similar objects together based on the likeness of their morphologies. The Predict algorithm attempts to predict the most appropriate model class for unclassified objects within a segment. These tools aid the user to improve the speed of model class population. All images were ultimately assigned to the respective ground truth model classes after visually examining the suggestions provided by the cluster and predict algorithms. Using these tools, all 758 base population objects were assigned to the three model classes—147 BNEs, 350 doublets, and 261 cells with irregular nuclear morphology. The AAI software then randomly split these objects into training, validation, and test sets using an 80/10/10 ratio, with the validation and test sets remaining unseen by the CNN during training^14,38^. Model training was completed when accuracy on the validation and training datasets converged, requiring 46 epochs. As described in our previous work^30^ objects classified as double nucleated (2N) images based on the spot count feature appeared to contain BNEs, doublets, and cells with irregular nuclear morphology. To analyze all remaining patient and healthy control data using the model, the 2N population in all remaining data files were loaded into the AAI software. The trained model was used to classify cellular images in the ProE, BasoE, and PolyOrthoE subpopulations into either BNEs, doublets, or cells with irregular nuclear morphology. Finally, all data files from patients and controls were updated to incorporate the classified objects from the AI model, enabling subsequent evaluation, including visual confirmation, to validate the classification outcomes.

### Manual verification of FBML and AAI classified BNEs

Individual images of FBML and AAI classified ProE, BasoE, and PolyOrthoE BNEs were manually inspected to visually evaluate accuracy of both techniques (Supplemental Fig. S5). To accomplish this, BF, CD235a, and DNA imagery of each individual cell were carefully reviewed to categorize true and false positive BNEs from both methods across the full data set. A total of 1910 BNEs were examined (FBML: n = 773; AAI n = 1137). To further evaluate the performance of the two algorithms, the number of false negative (FN) BNEs was quantified by summing the number of visually verified BNEs found within the doublets and cells with irregular nuclear morphology in ProE, BasoE, and PolyOrthoE populations. For FBML analysis Boolean algebra (Double nucleated AND NOT Doublets AND NOT BNEs) was used to define ProE, BasoE, and PolyOrthoE subset with irregular nuclei. A total of 1166 doublets (FBML: n = 547; AAI n = 619) and 4044 erythroblasts with irregular nuclei (FBML: n = 2245; AAI n = 1799) were validated. Moreover, all 2N populations (n = 3533) were assessed separately to establish manually classified BNE populations for all patients and healthy controls. Visually verified BNEs were identified and hand-tagged, resulting in the creation of manual ProE BNE, manual BasoE BNE, and manual PolyOrthoE BNE subpopulations. Individual object numbers from manually identified BNEs were then cross-referenced with object numbers for FBML and AAI identified BNEs. Venn diagrams were constructed to represent the relationship among the three sets of BNE object numbers identified by AAI, FBML, and manual classification in ProE, BasoE, and PolyOrthoE subsets form healthy volunteers (normal BM (NBM)), MDS patients, ICUS/CCUS, and non-MDS patients. These diagrams were generated using data derived from the summation of BNE counts identified by AAI, FBML, and manual classification. All cells were scored by the same observer to exclude inter-observer variability. While conducting manual scoring the observer was blinded against the FBML and AAI scoring results.

### Statistical analyses

The statistical analysis involved comparison of continuous variables between NBM and patients with MDS using the Mann–Whitney *U* non-parametric test. A significance level of p < 0.05 was used to determine statistical significance. All statistical calculations were performed using GraphPad Prism version 10 software (GraphPad Software, La Jolla, California). All BNE fractions are given with one decimal.

## Results

### Development of a deep-learning model utilizing Amnis^®^ AI (AAI)

**Figure 3:**
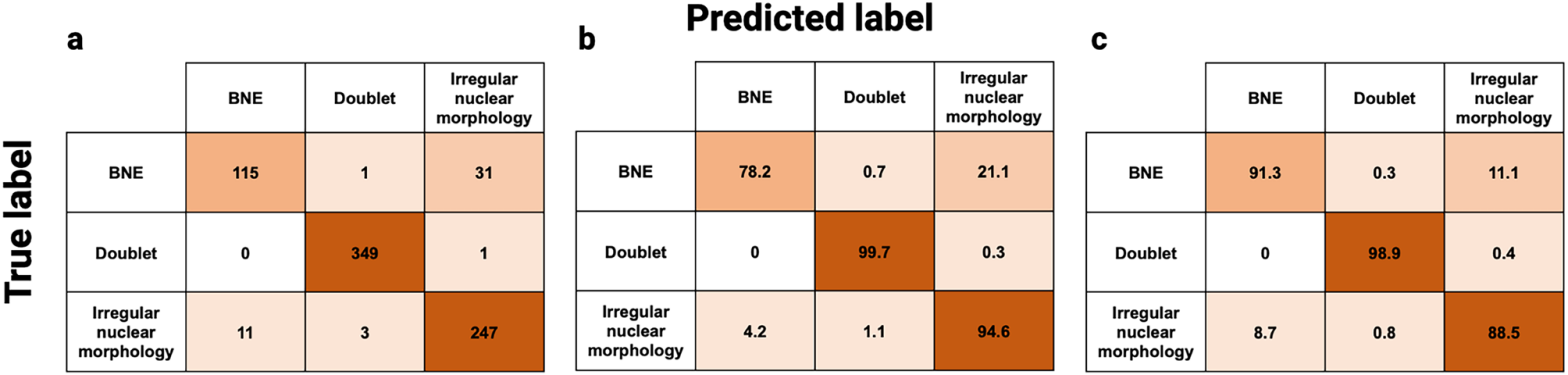
Confusion matrices and evaluation metrics. Three-class single-cell CNN classification using BF, CD235a, and DNA channels. Confusion matrices were generated for three categories using a dataset of 758 base population cells pooled from 7 MDS patients. (**a**) Class count numbers presenting the relationship between true and predicted class label, (**b**) row-wise normalization of class percentages emphasizing the sensitivity/recall (i.e., TPR: (TP/(TP + FN)) metrics for each individual class, and (**c**) column-wise normalization of class percentages emphasizing the precision (i.e., TP/TP + FP) of the model for each class. Created with BioRender.com.

To achieve accurate identification and quantification of dysplastic BNEs within BM samples from patients with MDS, it is pivotal to effectively differentiate them from both doublets and single nucleated erythroblasts exhibiting irregular nuclear morphological characteristics (Fig. 1). By employing the workflow illustrated in Fig. 2, we conducted training of an CNN algorithm that categorized candidate BNE erythroblasts into three classes: (i) BNEs, (ii) doublets, and (iii) erythroblasts with irregular nuclear morphology. After the training process, the AAI software provided common accuracy metrics used in artificial intelligence algorithms including precision, recall, and F1 score. Across model classes, the statistics ranged from 60 to 100% with overall weighted average model F1 scores of 94.8%, 90.6%, and 85.0% for the training, validation, and testing datasets, respectively (Table 1). The true and predicted class values from the experiment were recorded in confusion matrices (Fig. 3). The model demonstrated an accuracy of 94.3%, calculated as the sum of true positives (TPs) and true negatives (TNs) divided by the total count, signifying its overall effectiveness in predicting BNEs within the experimental dataset. Furthermore, it exhibited a specificity, or true negative rate (TN/TN + FP), of 98.2%, highlighting the model’s precision in accurately classifying objects as non-BNEs in 98.2% of the cases when they are not BNEs. In other words, misclassification of non-BNEs as BNEs was infrequent, occurring at a rate of only 1.8% and only erythroblasts with irregular nuclear morphology were mislabeled as BNEs (Fig. 3). The model demonstrated a sensitivity of 78.2%, underscoring the frequency with which the model correctly recalled true BNEs from the dataset. The reduced sensitivity was linked to misclassification of BNEs as erythroblasts exhibiting irregular nuclear morphology, implying that 21.1% of genuine BNEs in the dataset were incorrectly labeled as having a single irregular nucleus. Regarding the reliable identification of positive cases, the model exhibited strong precision (TP BNEs/predicted BNEs), accurately classifying BNEs as such in 91.3% of instances. Translated into a clinical context, designating an event as a BNE signifies a high level of confidence in its correctness. Nevertheless, a portion of actual BNEs may be incorrectly categorized as erythroblasts with irregular nuclear morphology, potentially leading to an underestimation of the BNE count.

**Table 1:** Accuracy statistics for the training, validation, and testing datasets used in the AAI BNE model.

Model class	Training data				Validation data				Testing data			
	Objects	Precision (%)	Recall (%)	F1 (%)	Objects	Precision (%)	Recall (%)	F1 (%)	Objects	Precision (%)	Recall (%)	F1 (%)
BNE	117	94.1	81.2	87.2	15	84.6	73.3	78.6	15	75.0	60.0	66.7
Doublets	234	100.0	99.6	99.8	35	97.2	100.0	98.6	35	92.1	100.0	95.9
Irregular nuclear morphology	209	89.8	97.1	93.3	26	85.2	88.5	86.8	26	80.8	80.8	80.8
Overall weighted average	560	95.0	94.8	94.8	76	90.6	90.8	90.6	76	84.9	85.5	85.0

### Performance of FBML and AAI for BNE quantification

**Figure 4:**
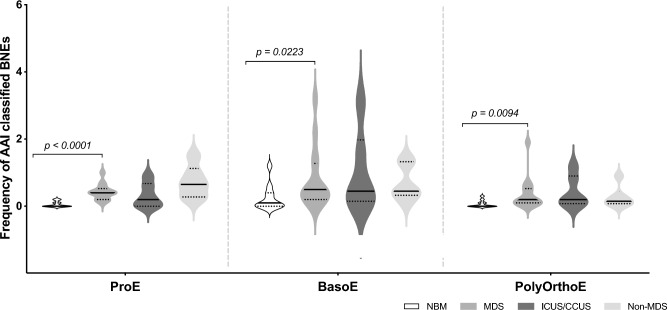
Frequency of BNEs in dyserythropoietic and control BM. Violin plot representation of BNE percentages among ProE, BasoE, and PolyOrthoE quantified by AAI. Groups of MDS patients (n = 14), ICUS/CCUS patients (n = 6), non-MDS patients (n = 6), and healthy controls (n = 11) are indicated by color, medians are highlighted by a solid line, and interquartile ranges are visualized by broken lines. Mann–Whitney *U* non-parametric test was used for comparison of healthy controls and MDS patients. ProE: p < 0.0001; BasoE: p = 0.0223; PolyOrthoE: p = 0.0094.

**Figure 5:**
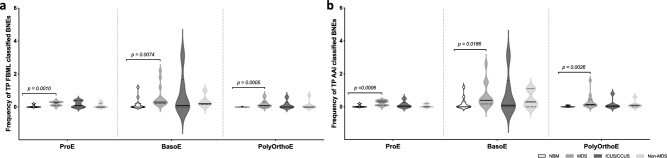
Frequency of true positive BNEs in dyserythropoietic and control BM following manual verification of individual BNE populations. Violin plot representation of BNE percentages among ProE, BasoE, and PolyOrthoE quantified by (**a**) FBML and (**b**) AAI. All BNE populations were manually inspected for visual verification and identification of true positive and false positive BNEs, respectively. Groups of MDS patients (n = 14), ICUS/CCUS patients (n = 6), non-MDS patients (n = 6), and healthy controls (n = 11) are indicated by color, medians are highlighted by a solid line, and interquartile ranges are visualized by broken lines. Mann–Whitney *U* non-parametric test was used for comparison of healthy controls and MDS patients. FBML ProE: p = 0.0010; FBML BasoE: p = 0.0074; FBML PolyOrthoE: p = 0.0005. AAI ProE: p = 0.0006; AAI BasoE: p = 0.0186; AAI PolyOrthoE: p = 0.0026.

The BNE frequencies in BM samples of healthy controls, patients with MDS, patients with ICUS/CCUS, and non-MDS patients were determined using AAI (Fig. 4). We have previously demonstrated that FBML could be used for enumeration of BNEs and found a significantly higher frequency in BM samples from MDS patients compared to healthy BM^30^. Here, we demonstrate that the AAI approach showed good agreement with the FBML approach in scoring BNE frequencies. Notably, significant increases in the number of BNEs were observed in MDS BM samples compared to healthy BM controls at all maturation stages (ProE: *p* < 0.0001; BasoE: *p* = 0.0223; PolyOrthoE: *p* = 0.0094) (Fig. 4). Despite the overall agreement between the two methods, we observed that the AAI method displayed a tendency towards identification of higher BNE numbers for individual MDS patients and controls—most visible for MDS patients and non-MDS patients (Supplemental Fig. S1) and leading to statistically significant increase in MDS BNE frequency at the BasoE maturation stage (*p* = 0.0223) (Fig. 4). We conducted a meticulous manual review of the BNE populations classified by both FBML and AAI. This involved visual examination and comparison of BF, CD235a, and DNA images of each individual cell (FBML: n = 773; AAI n = 1137). Upon this evaluation, the confirmed counts of TP BNEs revealed a decrease in the number of valid BNEs. Despite lower actual metric values, we consistently observed an increase in BNE frequencies (Fig. 5) and counts (Supplemental Fig. S2) in BM samples obtained from patients with MDS across all stages of maturation for both classification strategies. Moreover, when assessing MDS patients with BNE frequencies above the median value, both methods identified the same set of patients (data not shown). The nuclei of false positive (FP) BNEs displayed irregular shape and/or elongation (Supplemental Fig. S3a) and a notably increased number of FP BNEs was evident in BM samples obtained from patients with MDS, ICUS/CCUS, and non-MDS patients compared to healthy controls (Supplemental Fig. S3b–d). The healthy control group exhibited consistently low FP (Supplemental Fig. S3b–d) and false negative (FN) counts (Supplemental Fig. S4), establishing a reliable baseline for the implementation of an approach emphasizing deviations from the normal state. Specifically, when employing the FBML approach, only two out of 33 erythroblast subsets exhibited FP BNEs, with a median FP number of 1.5 (range 1–2). In contrast, the AAI method detected FP BNEs in eight subsets, with a median FP number of 2.0 (range 1–12) (Supplemental Fig. S3b–d). On the contrary, AAI showed a reduced incidence of FN BNEs (Supplemental Fig. S4), detecting them in eight out of 33 erythroblast subsets (median count: 1; range: 1–4) whereas FBML revealed FN BNEs in 11 out of 33 subsets, again with a median count of 1 (range: 1–5). Accordingly, despite not representing genuine BNEs, the abnormal nuclear shape and staining pattern of the FP BNEs in MDS, ICUS/CCUS, and non-MDS BM appeared different-from-normal, potentially reflecting a pathological condition within the BM.

**Figure 6:**
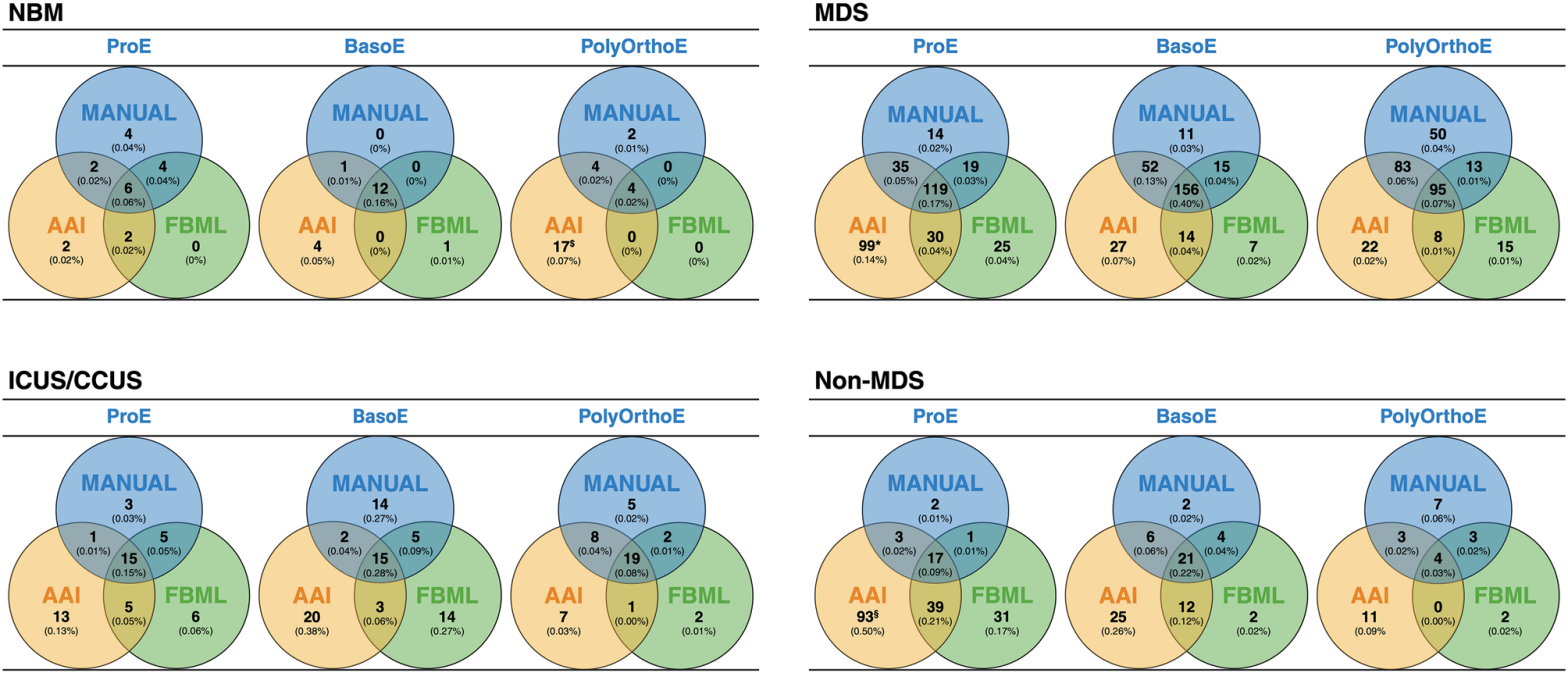
Venn diagrams. Graphic illustration of shared subsets and intersections between three sets of BNE object numbers identified manually, by AAI, and by FBML in ProE, BasoE, and PolyOrthoE subsets from NBM (n = 11), MDS patients (n = 14), ICUS/CCUS patients (n = 6), and non-MDS patients (n = 6). ^$^12 FP PolyOrthoE BNEs were identified in one healthy donor. *A total of 37 out of 99 FP ProE BNEs were associated with a single MDS patient. ^§^Altogether 71 out of 93 FP ProE BNEs were concentrated in only two patients (50 and 21, respectively). Created with BioRender.com.

As a quality control measure, the cells identified as BNEs by AAI were also assessed for their identification by FBML and manual classification (Fig. 6). Venn diagrams were constructed to represent the relationship among these three sets of BNE object numbers and determine shared subsets and intersections between the three methods. At the general level, we observed agreement among the three methods. Specifically, in 7 out of 12 subsets (ProE and BasoE in NBM; ProE, BasoE, and PolyOrthoE in MDS; ProE and BasoE in ICUS/CCUS), the majority of classified objects were identified by all three methods. In the case of the remaining five subsets, the imagery of the BNEs exclusively recognized by AAI displayed irregular and elongated nuclear shapes, signifying the presence of FP BNE identifications. Moreover, in relation to the FP BNEs, it’s worth highlighting that among the MDS patients, a substantial fraction (37 out of 99) of the FP ProE BNEs were linked to a single MDS patient. In the non-MDS group, the majority of FPs ProE BNEs (71 out of 93) were concentrated in just two patients (50 and 21 FP BNEs), while the remaining 22 FPs were distributed among the other four patients. A similar pattern emerged in the case of NBM at the PolyOrthoE stage, with 12 FPs identified in one donor and the remaining five FPs spread among three other donors. The Venn diagrams reveal that the majority of the cells identified through manual scoring were also correctly scored by either AAI or FBML. Notably, in MDS patients the subsets identified through both AAI and manual classification consistently contained a larger number of BNEs compared to the subsets identified through FBML and manual classification. In the context of NBM, ICUS/CCUS, and non-MDS patients, manual identification, when combined with either AAI or FBML, displayed a comparable agreement in terms of cell counts, albeit with some variability in the specific cells recognized by AAI and FBML.

## Discussion

The diagnosis of MDS is inherently challenging when relying on manual examination of BM slides, primarily due to the potential errors caused by smear quality and staining, inter-scorer variability, limited cell counts, and complexities associated with assessing subtle dysplastic changes^8,40,41^. As seen in other fields, AI has emerged as a valuable supplementary diagnostic tool^42–47^, holding the potential to benefit MDS diagnostics as well. While AI may offer significant assistance in streamlining laboratory workflows and serve as an effective screening tool, interpretation should be performed with care and individual model should be validated according to best practice within the field to ensure accuracy and reliability. Indeed, in our recent work, we demonstrated the capability of FBML in assisting the identification of dysplastic BNEs^30^. Nevertheless, while FBML proved effective in discriminating subtle morphologies, its implementation presented challenges owing to the inherent rigidity of mathematical features and the demand for specialized software expertise required for advanced IFC image analysis. Building upon this, we have now investigated the potential of employing a CNN algorithm for BNE quantification. The use of CNNs departs from the rigidity of feature-based analysis that relies on the combination of masks and mathematical features to differentiate populations. Rather, CNNs harness the abundant morphological and spatial information in individual images to emphasize specific, nuanced characteristics within an image set. Using an input of only 758 total objects, the CNN within the AAI software was able to generate a model for classification of BNEs, doublets, and irregularly shaped nuclei with high accuracy (94.3%). This strength has been demonstrated previously in other work where rare event characterization and quantification are at a premium^14,38^ and is realized here because CNNs can identify subtle morphologies in the imagery that are difficult to exploit using feature-based methods (e.g., bright CD235a expression at the boundary of a doublet image and the lack of nuclear circularity in an image with irregular morphology). Moreover, the time savings of an AI-based workflow compared to traditional feature-driven strategies are substantial. In IDEAS^®^ significant time was required to individually hand-tag the few hundred ground truth images and then determine an optimal gating strategy to identify true BNEs. However, with assistance from the Cluster and Predict algorithms in the AAI software, all loaded images were rapidly assigned to the appropriate ground truth model classes with ease using the GUI. All that is required from the user is to confirm that images are being assigned to the correct model class. Furthermore, once a model has been trained, multiple files can be analyzed by the CNN in just a few minutes as these algorithms can be rapidly applied to classify new data. Conversely, batch processing data in IDEAS^®^ can take several hours due to feature computation requirements. We employed seven out of the 37 available data files from our cohort for CNN model training. This constitutes 18.9% of the dataset, and given the rarity of BNEs, it was essential to ensure an adequate number of TP events to achieve high accuracy in the AAI software. The model demonstrated challenges in distinguishing between BNEs and erythroblasts with irregular nuclear morphology, as indicated by its modest recall rate (78.2%), implying that it couldn’t accurately identify all BNEs. This might result from subtle morphological differences between BNEs with closely positioned nuclei and erythroblast with irregularly shaped nuclei, which posed challenges in effectively distinguishing these two populations. Nonetheless, the CNN model displayed a high precision rate, signifying that when the model classified a cell as a BNE, there was a probability exceeding 90% that it indeed was a genuine BNE.

Overall results obtained with the CNN model were statistically comparable to the results obtained with FBML. Both methods identified a significant increase in the number of BNEs in MDS BM samples compared to healthy controls (Fig. 5), however the AAI method tended to identify higher BNE frequencies and numbers, particularly in MDS and non-MDS patients (Figs. 5, 6, and Supplemental Fig. S1), even after corrections for FP BNEs (Supplemental Fig. S2). Due to variations in the BNE quantification workflow (Supplemental Fig. S5), differences in the numbers of BNEs, FP BNEs, and FN BNEs classified by FBML and AAI could be expected. As CNN algorithms are more robust for identifying challenging morphology when compared to feature-based methods, a larger number of candidate BNE images were identified by AAI classification. Indeed, visual inspection of the imagery validated exclusion of genuine BNEs by the True 2N classifier, resulting in an increased number of FN BNEs for FBML classification (Supplemental Fig. S4). We observed that FN BNEs classified by both FBML, and the CNN, were classified as images with irregular nuclear morphology. This indicates that both methods can robustly differentiate BNEs from doublets. However, the identification of BNEs by either method breaks down when the regularity or irregularity of the nuclear morphology is challenging. In the context of rare event identification and quantification a high FN rate may underestimate disease severity, while a high FP rate may profoundly impact diagnosis accuracy. Essentially, the healthy control group exhibited consistently low FP and FN rates, establishing a robust baseline (Supplemental Figs. S3b–d and S4). This observation was pivotal for implementation of a different-from-normal approach centered on deviations from the normal state. The comprehensive identification of an increased number of genuine BNEs using the AAI method is essential to reduce the FN rate and prevent underestimating abnormalities in a clinical setting. Conversely, the increased FP rate could notably contribute to the perception that a patient has an elevated number of BNEs. However, FP BNEs were primarily identified in patients with known alterations or irregularities in their red blood cell production, or patients who were referred for evaluation due to persistent low red blood cell counts or issues related to red blood cell production, i.e., MDS, ICUS/CCUS, and non-MDS patients (Supplemental Fig. S3b–d). In other words, erythroblast maturation was disturbed in these patients, which may result in distinct changes in nuclear morphology, including irregularities, that might be mistakenly identified as true BNEs. These factors may explain the presence of a higher number of FP BNEs in MDS, ICUS/CCUS, and non-MDS patients compared to healthy individuals and in the context of a different-from-normal approach, everything not conforming to normality might be classified as abnormal. The incorrect classifications observed in certain patients and controls could result from various factors, including biological variations, quality and intensity of the DNA stain, crosstalk of the CD71 image due to suboptimal spectral compensation, and variations in nuclear morphology (e.g., size, shape, and staining accessibility). Effectively addressing these factors is essential, though challenging, particularly given the high parameter requirements for phenotypic analysis and the inherent diversity between patients.

In a diagnostic context, precise quantification of BNEs holds significant importance, highlighting the necessity for a robust classification model. In the present case, the occurrence of FP and FN observed by AAI classification could likely be ascribed to limitations in the training data. To enhance AI model accuracy, augmenting the data related to challenging classes can help the AI system in capturing the class nuances and improve future classifications^14,38,39,48^. Given the relatively small dataset comprising just 758 objects used for CNN model training, introducing a more extensive image dataset, potentially encompassing several thousand images per class, may substantially improve model performance. Emphasis should be placed on augmenting the classes of cells with irregular nuclear morphology and BNEs that posed classification difficulties. Moreover, expanding the range of classification categories to include a category for uncertain cases, in addition to BNEs, doublets, and cells with irregular nuclear morphology, could be beneficial. Yet, this is currently not possible with the AAI software due to the requirement of providing ground truth data to introduce a new class. The AAI software package offers a user-friendly interface, that eliminates the need for prior programming expertise, making it accessible to a broader community of researchers. However, the intuitive model construction comes with limitations as the software lacks customization options. Users have limited control over crucial training aspects, such as the number of layers, data partitioning, and probability threshold values, all of which are pre-determined by the software. For now, each classified object is assigned a classification probability, with higher values indicating the likelihood of its membership in a specific class. The introduction of a classification threshold could prove valuable, as it would necessitate that the classification probability exceeds this threshold for an object to be assigned to a specific class. Objects not meeting this threshold would be categorized as unknown. Furthermore, the utilization of multiple algorithms for data analysis may enable a more comprehensive investigation of a dataset, potentially enhancing the depth and precision of data analysis. An alternative approach to the AAI software package, is presented in the form of the open-source software DeepFlow architecture^49^. Studies have demonstrated its ability to notably enhance classification accuracy in various contexts, including phenotypic cell cycle analysis^49^. In this regard, it has superior performance compared to traditional machine learning methods that rely on feature extraction from cell images^50^. Additionally, DeepFlow has demonstrated effectiveness in classifying BM cells from patients with acute lymphoblastic leukemia^32^ and assessing morphological changes in stored red blood cells^21,51^. By combining both strategies—providing additional relevant truth data and employing alternative algorithms—classification errors may be reduced while the overall AI classification accuracy may be enhanced.

This study, combined with our prior publication^30^, highlights the advantage of using IFC for automated quantification of BNEs. Binucleation, a notable yet rare dysplastic feature in MDS, faces limitations in slide-based quantification due to the analysis of only a few hundred cells, implicating statistical power. By capturing thousands of cellular images using IFC, the probability of detecting rarely present BNEs significantly increases. This approach alleviates the need for the time-consuming and labor-intensive task of manual BNE counting, eliminates subjectivity, and reduces inter-observer variability. Importantly, IFC captures high-quality images of all key populations that can be scored through either feature-based analysis or deep-learning algorithms. Building on our different-from-normal approach, we have extended our previous finding to illustrate that the combination of IFC with an AI algorithm provides a more straightforward workflow compared to feature-based analysis, leading to detection of a greater number of genuine BNEs in patient samples. This approach serves as a powerful tool for assessing subtle and infrequent morphological features in MDS, potentially improving diagnostic precision. Collectively, the results presented in this study contribute to the body of existing literature, emphasizing the potential utility of IFC for clinical phenotyping, with the possibility of its translation into a diagnostic tool for clinical practice^21,32,34,52,53^.

### Supplementary Information

Supplementary Figures.

## Data Availability

The datasets generated during and/or analyzed during the current study are available from the corresponding author on reasonable request.

## Abbreviations

AAI: Amnis AI
BasoE: Basophilic erythroblast
BF: Brightfield
BM: Bone marrow
BNE: Binucleated erythroblast
CNN: Convolutional neural network
FBML: Feature based machine learning
FN: False negatives
FP: False positive
MDS: Myelodysplastic syndrome
2N: 2 Nuclei
NBM: Normal bone marrow from healthy volunteers
PolyOrthoE: Polyorthochromatic erythroblast
ProE: Proerythroblast
PE: R-Phycoerythrin
TP: True positives
TPR: True positive rate
