# Author Correction: Comparative analysis of feature-based ML and CNN for binucleated erythroblast quantification in myelodysplastic syndrome patients using imaging flow cytometry data

**DOI:** 10.1038/s41598-024-63712-6

**Published:** 2024-06-06

**Authors:** Carina A. Rosenberg, Matthew A. Rodrigues, Marie Bill, Maja Ludvigsen

**Affiliations:** 1 Department of Hematology, Aarhus University Hospital, Palle Juul‑Jensens Boulevard 35, C115, 8200 Aarhus C, Denmark; 2 Amnis Flow Cytometry, Cytek Biosciences, Seattle, WA USA; 3 Department of Clinical Medicine, Aarhus University, Aarhus, Denmark

Correction to: *Scientific Reports* 10.1038/s41598-024-59875-x, published online 23 April 2024

In the original version of this Article Matthew A. Rodrigues was incorrectly affiliated with ‘RareCyte, Inc., Seattle, WA, USA’. The correct affiliation is listed below.

Amnis Flow Cytometry, Cytek Biosciences, Seattle, WA, USA.

The original Article has been corrected.

