# Supplementary material for: Comparative analysis of feature-based ML and CNN for binucleated erythroblast quantification in myelodysplastic syndrome patients using imaging flow cytometry data
Source: Sci Rep. 2024 Apr 23;14:9349. doi: 10.1038/s41598-024-59875-x (PMC11039460; doi:10.1038/s41598-024-59875-x)
Supplement: Supplementary file 1 — Supplementary Figures. [file 41598_2024_59875_MOESM1_ESM.pdf]

SUPPLEMENTARY DATA

Comparative Analysis of Feature-Based ML and CNN for Binucleated Erythroblast Quantification in Myelodysplastic Syndrome Patients using Imaging Flow Cytometry data

Carina A. Rosenberg[1]; Matthew A. Rodrigues[2,3]; Marie Bill, MD PhD[1,4]; and Maja Ludvigsen, MSc PhD[1,4]

[1]Department of Hematology, Aarhus University Hospital, Aarhus, Denmark
[2]Amnis Flow Cytometry, Cytek Biosciences, Seattle, WA, USA
[3]RareCyte, Inc., Seattle, WA, USA
[4]Department of Clinical Medicine, Aarhus University, Aarhus, Denmark

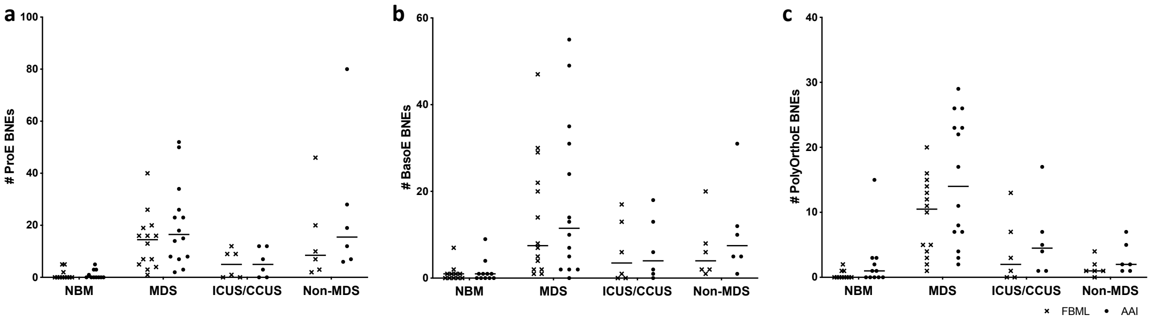

Supplementary Figure S1. Numbers of BNEs in BM samples from MDS patients and controls. Scatter plots illustrating the numbers of BNEs identified by FBML or AAI for MDS patients (n=14), ICUS/CCUS patients (n=6), non-MDS patients (n=6), and healthy controls (n=11). Medians are highlighted by a solid line. (**a**) ProE, (**b**) BasoE, and (**c**) PolyOrthoE.

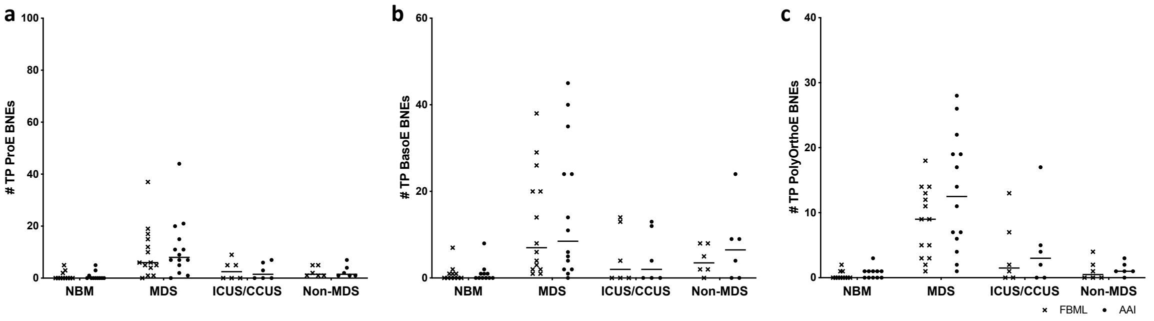

Supplementary Figure S2. Numbers of TP BNEs in BM samples from MDS patients and controls. Scatter plots depicting the numbers of visually confirmed TP BNEs in datasets originating from FBML and AAI classification for MDS patients (n=14), ICUS/CCUS patients (n=6), non-MDS patients (n=6), and healthy controls (n=11). Medians are highlighted by a solid line. (**a**) ProE, (**b**) BasoE, and (**c**) PolyOrthoE.

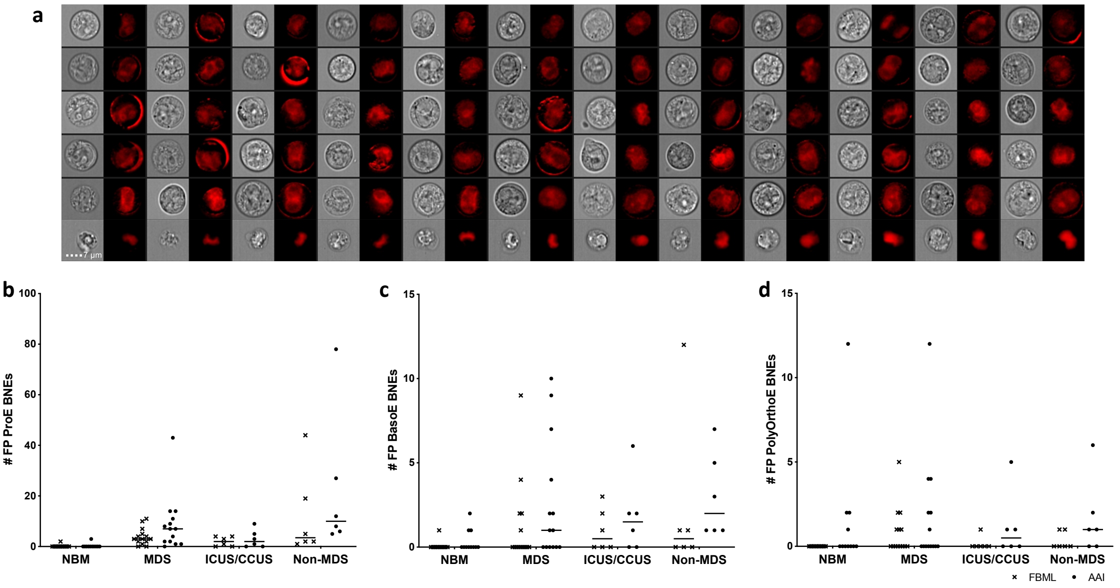

Supplementary Figure S3. FP BNEs in BM samples from MDS patients and controls. (**a**) Representative DNA imagery illustrating FP BNEs with irregular and/or elongated shape. Scatter plots displaying the numbers of visually confirmed FP BNEs in datasets originating from FBML and AAI classification for MDS patients (n=14), ICUS/CCUS patients (n=6), non-MDS patients (n=6), and healthy controls (n=11). Medians are highlighted by a solid line. (**b**) ProE, (**c**) BasoE, and (**d**) PolyOrthoE.

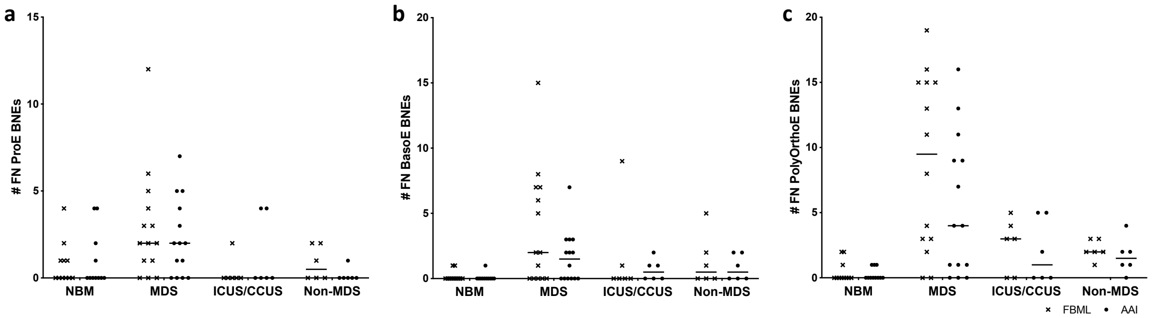

Supplementary Figure S4. FN BNEs in BM samples from MDS patients and controls. Scatter plots displaying the numbers of visually confirmed FN BNEs in datasets originating from FBML and AAI classification for MDS patients (n=14), ICUS/CCUS patients (n=6), non-MDS patients (n=6), and healthy controls (n=11). Medians are highlighted by a solid line. (**a**) ProE, (**b**) BasoE, and (**c**) PolyOrthoE.

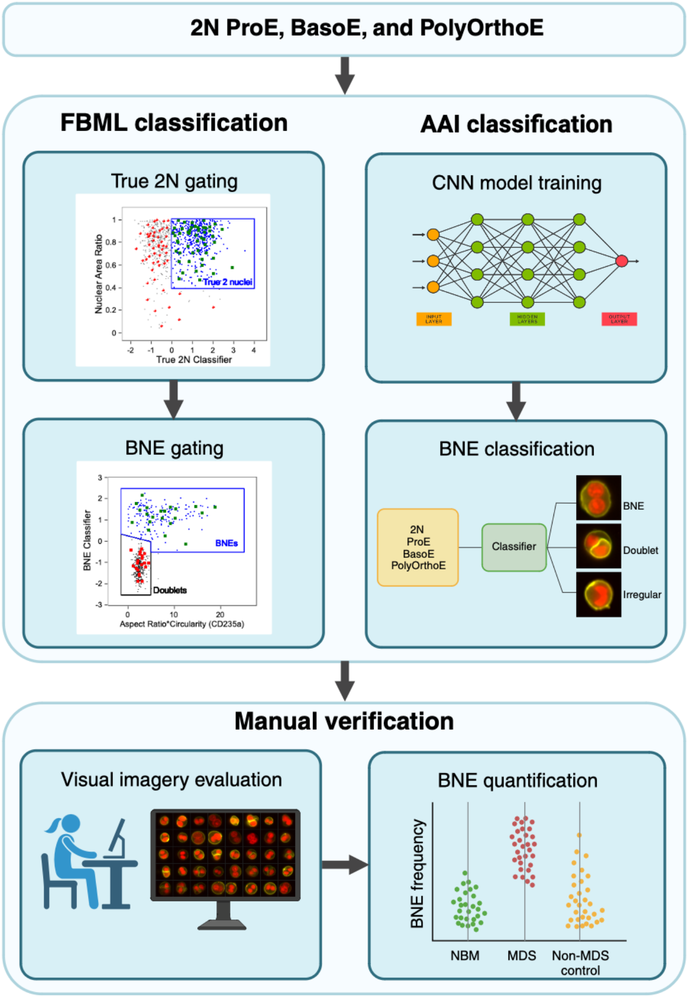

Supplementary Figure S5. Schematic BNE quantification workflow. Graphic illustration of the workflow used for BNE quantification by FBML classification (left) and AAI classification (right). For both approaches IDEAS-gated 2N ProE, BasoE, and PolyOrthoE subpopulations were used as base populations. Candidate BNEs gated by FBML or classified by AAI were manually verified by visual inspection of the imagery. Created with BioRender.com
